# The G9a Histone Methyltransferase Inhibitor BIX-01294 Modulates Gene Expression during *Plasmodium falciparum* Gametocyte Development and Transmission

**DOI:** 10.3390/ijms20205087

**Published:** 2019-10-14

**Authors:** Che Julius Ngwa, Meike Jutta Kiesow, Lindsey Marie Orchard, Afia Farrukh, Manuel Llinás, Gabriele Pradel

**Affiliations:** 1 Division of Cellular and Applied Infection Biology, Institute of Zoology, RWTH Aachen University, 52074 Aachen, Germany; kiesow@bio2.rwth-aachen.de (M.J.K.); afiafarrukh10@gmail.com (A.F.); pradel@bio2.rwth-aachen.de (G.P.); 2 Department of Biochemistry and Molecular Biology & Center for Malaria Research, The Pennsylvania State University, University Park, PA 16802, USA; lma20@psu.edu (L.M.O.); manuel@psu.edu (M.L.)

**Keywords:** histone methylation, gene expression, malaria, gametocytes, transmission

## Abstract

Transmission of the malaria parasite *Plasmodium falciparum* from the human to the mosquito is initiated by specialized sexual cells, the gametocytes. In the human, gametocytes are formed in response to stress signals and following uptake by a blood-feeding *Anopheles* mosquito initiate sexual reproduction. Gametocytes need to fine-tune their gene expression in order to develop inside the mosquito to continue life-cycle progression. Previously, we showed that post-translational histone acetylation controls gene expression during gametocyte development and transmission. However, the role of histone methylation remains poorly understood. We here use the histone G9a methyltransferase inhibitor BIX-01294 to investigate the role of histone methylation in regulating gene expression in gametocytes. In vitro assays demonstrated that BIX-01294 inhibits intraerythrocytic replication with a half maximal inhibitory concentration (**IC_50_**) of 13.0 nM. Furthermore, BIX-01294 significantly impairs gametocyte maturation and reduces the formation of gametes and zygotes. Comparative transcriptomics between BIX-01294-treated and untreated immature, mature and activated gametocytes demonstrated greater than 1.5-fold deregulation of approximately 359 genes. The majority of these genes are transcriptionally downregulated in the activated gametocytes and could be assigned to transcription, translation, and signaling, indicating a contribution of histone methylations in mediating gametogenesis. Our combined data show that inhibitors of histone methylation may serve as a multi-stage antimalarial.

## 1. Introduction

The tropical disease malaria still remains a global health burden with about 219 million infections and almost half a million deaths recorded in 2017 [1]. The disease is caused by intracellular parasites of the genus *Plasmodium* with *Plasmodium falciparum*, the causative agent of malaria tropica, being the most severe form of malaria. The transmission of *P. falciparum* from the human host to the *Anopheles* vector is mediated by sexual cells, the gametocytes. These develop inside the human red blood cells and, upon uptake by the blood-feeding *Anopheles* mosquito, rapidly transform into gametes [2,3]. The gametocytes are the only stages that are able to initiate sexual reproduction in the mosquito, therefore they are vital for the spread of the disease. The intraerythrocytic development of gametocytes as well the activation in the mosquito midgut are supported by a well-coordinated sequence of gene activation and silencing events [4,5,6]. These changes in the gene expression pattern are mandatory to prepare the malaria parasite for transmission from the human to the insect host.

Research over the past decade has demonstrated a pivotal role of epigenetics-mediated regulation of gene expression in eukaryotes. A major part of epigenetic control involves histone acetylations and methylations, which are mediated by specialized histone “writers”, the histone acetyltransferases (HATs) and histone methyl transferases (HMTs), as well as histone “erasers”, the histone deacetylases (HDACs) and histone demethylases (HDMs), respectively. HATs promote DNA accessibility resulting in gene transcription while HDACs inhibit DNA accessibility, thereby preventing gene transcription. HMTs on the other hand can either act as promotors or inhibitors of DNA accessibility, depending on the methylation site [7,8,9].

The *P. falciparum* genome encodes five plasmodial HDACs; i.e., HDAC1 and HDAC3, Hda2 and the two type III silent information regulators Sir2a and Sir2b [10,11,12] as well as four HATs, including MYST and PfGNC5 [13,14]. Furthermore, the genes encoding for ten SET (Su(var)3–9-′Enhancer of zeste-Trithorax)-domain-containing lysine-specific HMTs, termed SET1 to SET10 [15,16] and the three protein arginine methyltransferases PRMT1, PRMT5 and CARM 1 [17] have been identified. In addition, three demethylases, i.e., LSD1, JmjC1 and JmjC2, are encoded in the genome of *P. falciparum* [18].

To date, epigenetic regulation of gene expression in the malaria parasite has particularly been studied in the asexual blood stages during expression of virulence-associated var genes [9,19]. Approximately 60 var genes encode for the erythrocyte membrane protein *P. falciparum* erythrocyte membrane protein 1 (PfEMP1) in the *P. falciparum* blood stages [9,12,20,21,22,23]. Out of the 60 var genes, only one is expressed at a time, and this is associated to the pathogenesis and immune evasion by the parasite. The expression of var genes depends on epigenetic mechanisms with the active var gene assuming an euchromatic state due to acetylation of lysine 9 and tri-methylation of lysine 4 on histone 3 (H3K9ac and H3K4me3), respectively [24,25]. On the other hand, var gene silencing is associated with H3K9 and H3K36 tri-methylation (H3K9me3, H3K36me3), Sir2A, Sir2B and the class II HDAC PfHda2 [18,26,27,28,29].

Several recent studies further reported a crucial function of epigenetics-mediated gene regulation during sexual commitment, when the asexual blood stage parasites enter the sexual pathway to form gametocytes [9,30]. Sexual commitment is triggered by environmental signals [2,3]. In committed asexual blood stage parasites, stress and other unknown factors cause the removal of the histone H3K9me3 mark. The removal of this repressive epigenetic mark leads to the expression of an AP2-G transcription factor, which in consequence controls the expression of genes required for gametocyte formation [30,31,32,33]. In non-committed asexual blood stage parasites, heterochromatin protein 1 binds specifically to H3K9me3 to maintain the heterochromatin state, thereby hindering the expression of AP2-G, resulting in suppression of gametocyte commitment [34].

Despite increasing insights into the role of epigenetics during gametocyte commitment, little is known about such mechanism of gene regulation during gametocyte development. We previously showed by a chemical loss of function study using the HDAC inhibitor Trichostatin A (TSA) that acetylation-based histone modifications are vital for preparing the parasite for human-to-mosquito transmission [5]. In this follow-up study, we now report that the histone G9a HMT inhibitor BIX-01294 also modulates gene expression in gametocytes, thereby confirming a crucial contribution of histone methylation during malaria transmission.

## 2. Results and Discussion

### 2.1. P. falciparum SET-Domain-Containing HMTs Are Expressed in Gametocytes

**Figure 1:**
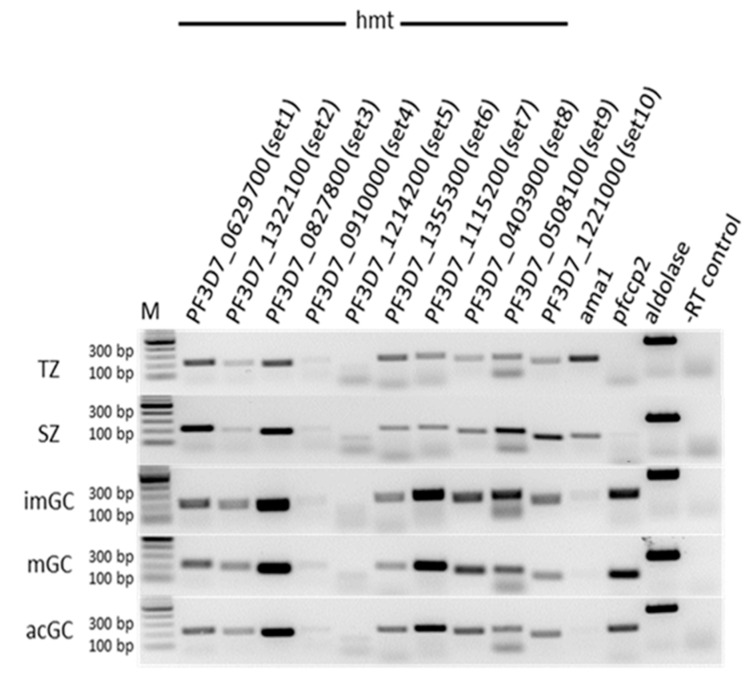
Transcript expression of the 10 SET-domain containing proteins (HMTs) in *P. falciparum*. Complementary DNA prepared from trophozoites (TZ), schizonts (SZ), immature (imGC), mature (mGC) and gametocytes at 30 min post-activation (aGC) were subjected to RT-PCR using gene-specific primers for HMTs of *P. falciparum*. The expression levels of *pfama1* and *pfccp2* were used to verify blood-stage and gametocyte-specific expression. Samples lacking reverse transcriptase (-RT) were used as controls for genomic DNA contamination. *pfaldolase* was used as a loading control.

To determine if the *P. falciparum* SET-domain-containing HMTs are expressed in gametocytes, we performed a semi-quantitative RT-PCR using RNA from immature and mature gametocytes as well as from gametocytes at 30 min post-activation (p.a.). Transcript from trophozoites and schizonts was used for comparison. High transcript levels for SET1, SET3 and SET7 to SET10 were observed in trophozoites and schizonts, and of SET1 to SET3 and SET6 to SET10 in immature, mature and activated gametocytes (Figure 1). For SET4 and SET5, only low transcript levels could be detected in any of the parasite stages. The stage-specificity of the isolated RNA was controlled by determining the transcripts of stage-specific genes, i.e., *pfama1* which encodes the apical membrane antigen 1 and *pfccp2* encoding the LCCL-domain protein 2. Transcript analysis for the housekeeping gene *pfaldolase* encoding the enzyme aldolase was also included as loading control and showed almost equal loading of cDNA samples. The absence of gDNA in the samples was confirmed by using a control lacking reverse transcriptase (Figure 1). These data are in accordance with transcriptomics data available at the PlasmoDB database in which the transcriptome of seven stages of the malaria parasite was analysed by RNA sequencing [6]. The high transcript expression of some SET-domain-containing HMTs in gametocytes indicate a possible role of histone methylation in the control of gametocyte gene expression. Primer specificity was also verified by diagnostic PCR on gDNA (Figure S1).

### 2.2. BIX-01294 Treatment Impairs P. falciparum Asexual Blood Stage Replication and Sexual Development

To investigate the role of histone methylation during development of the *P. falciparum* asexual and sexual blood stages, the effect of a commercially available HMT inhibitor BIX-01294 during intraerythrocytic replication was tested, using the gametocyte-producing *P. falciparum* NF54 strain. The inhibitor blocked asexual blood stage replication with an IC_50_ value of 13.0 ± 2.31 nM, as shown by the Malstat-based viability assay. These data are in accordance with previous reports on BIX-01294 inhibiting intraerythrocytic replication in both *P. falciparum* drug sensitive strain (3D7) and multi-drug resistant field isolates at nM ranges [35,36].

**Figure 2:**
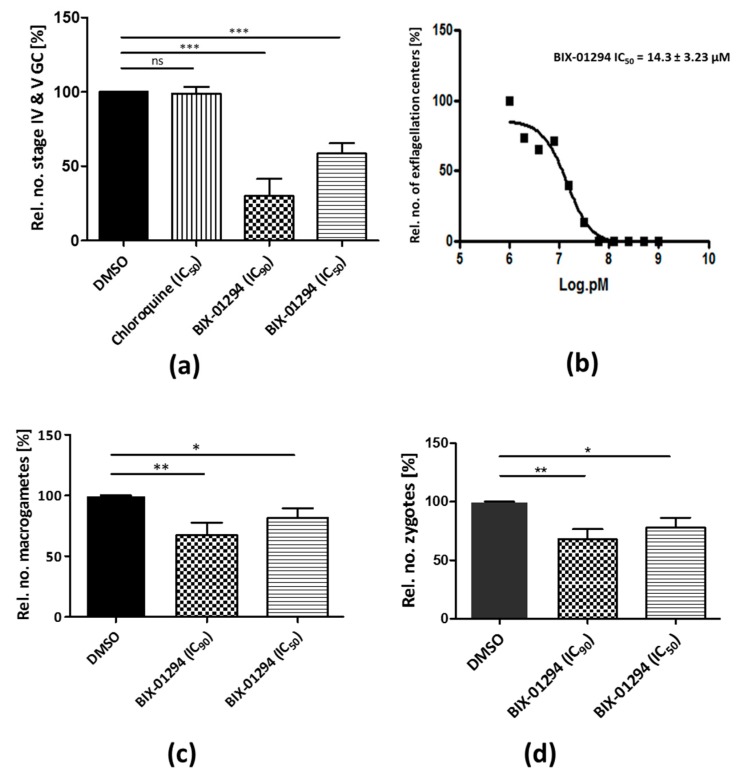
Effect of BIX-01294 on *P. falciparum* sexual development. (**a**) Effect of BIX-01294 on gametocyte (GC) development. BIX-01294 at IC_50_ or IC_90_ concentrations was added to stage II GC cultures for 48 h. Numbers of stage IV and V GC were determined in 1000 RBCs after 10 days. 1% vol. DMSO and chloroquine (16 nM) were used as negative controls (DMSO set to 100%); (**b**) effect of BIX-01294 on microgamete exflagellation. Mature GC cultures were pre-incubated with BIX-01294 in concentrations ranging between 1 mM to 1 µM for 15 min at 37 °C. Each sample was then transferred to RT and xanthurenic acid was added at a concentration of 100 µM for activation. After another 15 min, the numbers of exflagellation centers were counted in 30 optical fields in duplicate using a Leica DMLS microscope (Wetzlar, Germany) (400-fold magnification). The data (**b**) are representative of two independent experiments; (**c**,**d**) effect of BIX-01294 on macrogamete and zygote formation, respectively. Mature GC culture was incubated with BIX-01294 at IC_50_ or IC_90_ concentrations or 1% vol. DMSO for 1 h at 37 °C, activated with xanthurenic acid (XA) and cultured at RT for 30 min (macrogametes) or 12 h (zygotes). Detection was achieved by immunolabelling with anti-Pfs25 and counted in triplicate in 1000 RBCs. Results shown (for a, c and d) are combined from three independent experiments each performed in triplicate (mean ± SD). *p < 0.05; **p < 0.01; ***p < 0.001; ns, not significant; Student’s *t*-test.

We then determined the effect of the inhibitor on gametocyte development. To achieve this, a gametocyte stage II rich culture was treated with BIX-01294 at IC_50_ and IC_90_ concentrations or 1% vol. Dimethyl sulfoxide (DMSO; solvent control) for 48 h. The treated parasites were then cultured for another 8 d without inhibitor and the gametocytemia was analyzed every 2 d via staining with Giemsa. Treatment of early gametocytes with BIX-01294 significantly reduced the numbers of stage IV and V gametocytes formed on day 10 to 50.7 ± 3.95% and 30.0 ± 6.68% at IC_50_ and IC_90_ concentrations, respectively, as compared to DMSO controls (Figure 2a). While BIX-01294 treatment resulted in the killing of early gametocytes, it did not cause a delay in gametocyte maturation compared to the controls (Figure S2a–d). In addition, BIX-01294 treatment did not lead to any morphologic abnormalities in stage IV and V gametocytes as compared to controls (Figure S2e). The effect of BIX-01294 on male gamete formation was also tested by determining the ability of male gametocytes to exflagellate. BIX-01294 inhibited exflagellation with an IC_50_ value of 14.3 ± 3.23 µM (Figure 2b). To find out if BIX-01294 treatment affects macrogamete and zygote formation, mature gametocyte cultures were treated with BIX-01294 at IC_50_ and IC_90_ concentrations or 1% vol. DMSO (untreated control) for 1 h at 37 °C. After treatment, the gametocytes were activated with 100 µM xanthurenic acid (XA) and further incubated at RT for 30 min and 12 h for macrogametes and zygotes to be formed, respectively. The macrogametes and zygotes were then immunolabelled with anti-Pfs25 antibodies and counted in a total of 1000 erythrocytes in triplicate. Macrogamete formation was significantly reduced to 81.6 ± 4.59% and 67.5 ± 5.92% and zygote formation to 77.9 ± 4.93% and 67.9 ± 4.97%, at asexual blood stage IC_50_ and IC_90_ concentrations, respectively (Figure 2c,d). 

In support of these data, Malmquist and colleagues also showed a strong transmission blocking effect of BIX-01294 with microgametocytes being the most affected stage [36]. It is noteworthy that the inhibitory activity of BIX-01294 against the sexual stages was significantly stronger than reported earlier for the HDAC inhibitor TSA [5], indicating that histone methylations might play a prominent role in regulating gene expression during the sexual phase of the parasite.

### 2.3. Treatment of Gametocytes with BIX-01294 Slightly Affects H3K4me3 and H3K9me3

**Figure 3:**
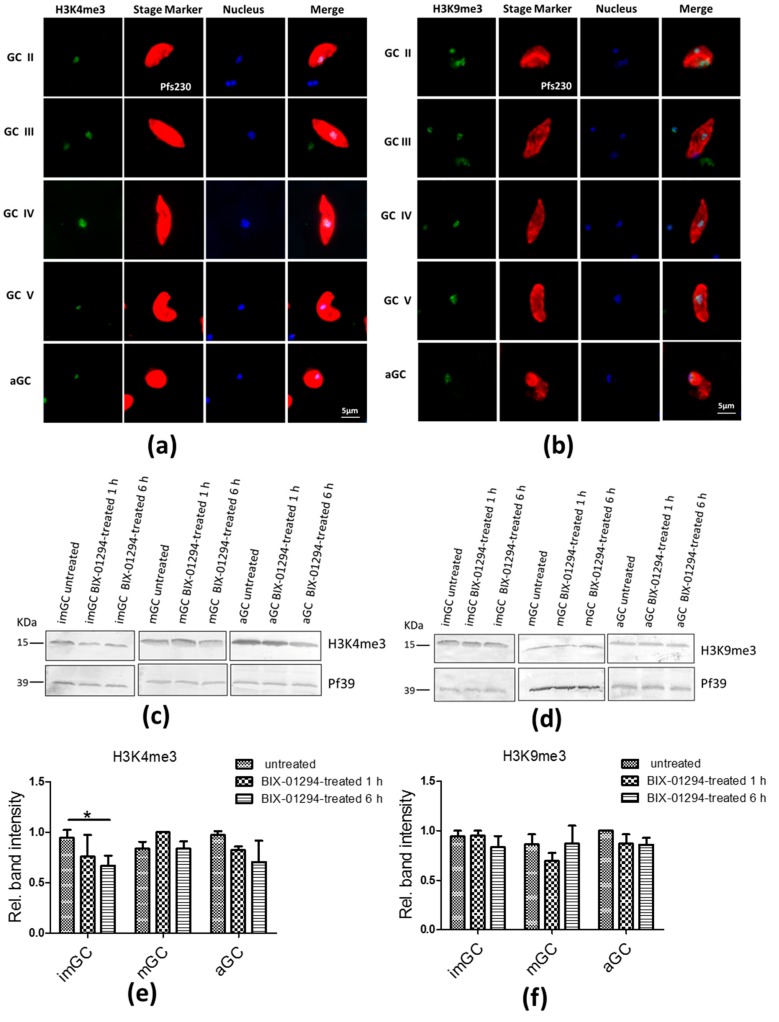
H3K4me3 and H3K9me3 levels following treatment of gametocytes with BIX-01294. (**a**,**b**) Detection of H3K4me3 and H3K9me3 marks in gametocytes by indirect immunofluorescence assays. Presence of H3K4me3 and H3K9me3 in gametocytes were detected in the different gametocyte stages (GC stage II-V) via immunolabelling using rabbit anti-H3K4me3 (**a**) and anti-H4K9me3 (**b**) (green). Gametocytes were highlighted with mouse antibodies against the gametocyte marker Pfs230 (red). Nuclei were highlighted by Hoechst nuclear stain 33342 (blue). Bar, 5 μm. (**c**,**d**) detection of H3K4me3 (**c**) and H3K9me3 (**d**) in BIX-01294 treated and untreated gametocytes by Western blotting. Lysates of immature (imGC) and mature gametocytes (mGC) following treatment with BIX-01294 at IC_90_ concentrations or 1% vol. DMSO (untreated) for 1 and 6 h at 37 °C were subjected to Western blot analysis using anti-H3K4me3 and anti-H3K9me3 antibodies; (**e**,**f**) quantification of H3K4me3 and H3K9me3 following BIX-01294 treatment of gametocytes. Histone methylation was quantified between BIX-01294 treated and untreated samples by measuring the band intensities following immunoblotting via ImageJ for three different experiments (**e**,**f**); values were normalized with band intensities of Pf39 used as loading control (set to 1). *p < 0.05).

BIX-01294 is an inhibitor of the HMT G9a, which mainly methylates H3K9 marks [37]. We thus aimed to investigate, if BIX-01294 affects H3K9me3 methylation, a repressive mark with important roles in var gene regulation and sexual commitment (see above). We further tested an effect of the inhibitor on H3K4me3 methylation, and an active mark also linked to var gene regulation [24,38]. Initially, we demonstrated by indirect immunofluorescence assay, using commercially available antibodies against H3K4me3 and H3K9me3, that both histone marks are present in the nuclei of gametocyte stages II to V as well as of activated gametocytes (Figure 3a,b). Normal rabbit serum (NRS) was used as negative control and showed no labelling in gametocytes (Figure S3).

Subsequently, we assessed if treatment of gametocytes with BIX-01294 affects the histone methylation marks. In this regard, enriched immature, mature and activated gametocytes were treated with BIX-01294 for 1 and 6 h at IC_90_ concentrations and lysates were prepared and the levels of H3K4me3 and H3K9me3 marks were determined by Western blotting. Immunoblotting confirmed the presence of both marks in immature, mature and activated gametocytes in the presence and absence of BIX-01294, running at the expected molecular weights of approximately 15 kDa for H3K4me3 and 17 kDa for H3K9me3 (Figure 3c,d). When the band intensities were measured by ImageJ (Version 1.48v, National Institutes of Health, Maryland, USA), significantly reduced levels of H3K4me3 marks following 6 h of BIX-01294 treatment were observed in immature gametocytes and an additional reduction in H3K4me3 and H3K9me3 was observed in activated gametocytes following 1 and 6 h BIX-01294 treatment (Figure 3e,f). It is noteworthy that no effect was observed for the H3K9me3 marks in immature and mature gametocytes (Figure 3e,f).

Our results show the presence of H3K4me3 and H3K9me3 marks in gametocytes. This is in conformity with previously conducted chromatin proteomics in asexual and sexual blood stages of *P. falciparum* [39]. It is noteworthy that BIX-01294 treatment particularly reduced H3K4me3 methylation, suggesting that in gametocytes the inhibitor might rather affect this active mark rather than impairing repression. Only the treatment of gametocytes with BIX-01294 for 6 h, however, revealed a significant effect on the H3K4me3 in immature gametocytes and a minor effect on activated gametocytes. The inability to detect highly significant changes in H3K4me3 and H3K9me3 methylation is probably due to the fact that the gametocytes were treated only for a short period of 6 h. For comparison, a significant reduction in H3K4me3 levels in the *P. falciparum* asexual blood stages could only be detected after 12 h treatment [35].

### 2.4. BIX-01294 Treatment Results in Modulation of Gene Expression During Gametocyte Development

The effect of BIX-01294 on gene expression in the different gametocyte stages was investigated by comparative microarray analysis. Immature, mature and activated gametocytes were treated with BIX-01294 at IC_90_ concentrations for 1 h or 6 h, and DMSO-treated parasites were used as control. The purity of the cell samples was confirmed via Giemsa smears. RNA was isolated and cDNA was synthesized and labelled. The labelled samples were then applied to a DNA Agilent microarray chip (Agilent Technologies,Santa Clara, USA) containing 5363 *P. falciparum* coding genes [40,41]. The transcript levels of genes greater or lower than 1.5-fold compared to the control for at least one of the two time-points combined with a consistent up- or downregulation for both time-points were used for further analysis [5].

**Figure 4:**
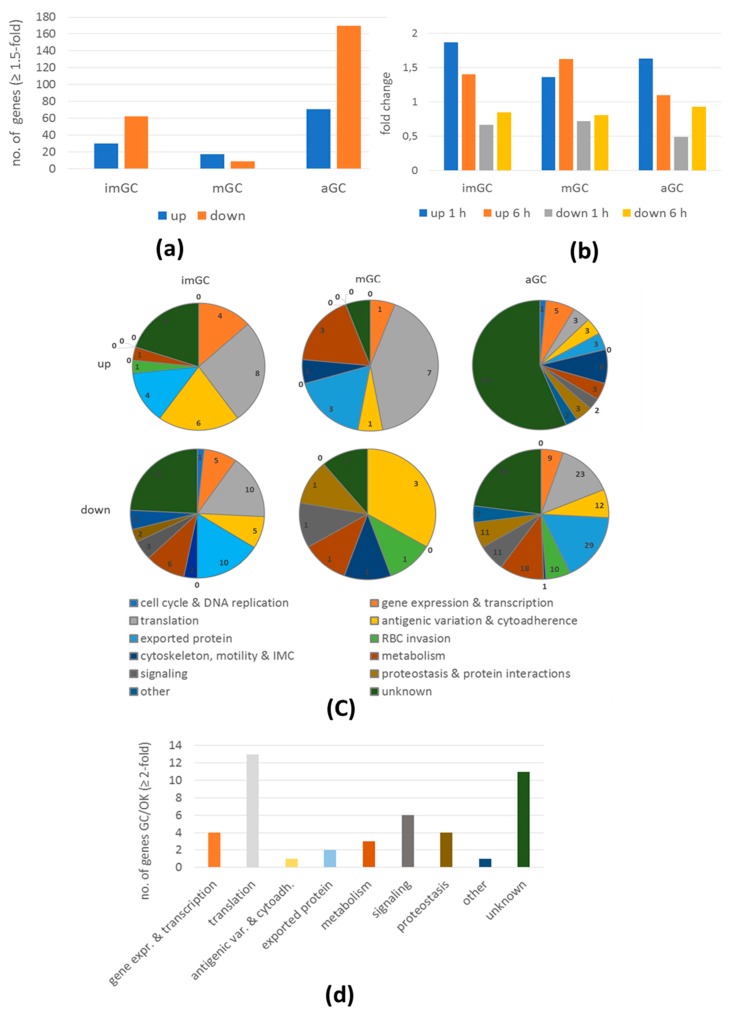
De-regulation of gene expression following BIX-01294 treatment of gametocytes. Immature (imGC) and mature (mGC) gametocytes as well as gametocytes at 1 h post-activation (aGC) were treated with BIX-01294 at IC_90_ concentrations or 0.5% vol. DMSO (untreated) for 1 and 6 h, total RNA was isolated and cDNA synthesized to be employed in microarray assays. Genes with a relative expression levels greater than 1.5-fold for at least one of the two time-points combined with a consistent up- or downregulation for both time-points were used for further analysis. (**a**) bar charts showing total up- and downregulated genes in imGC, mGC and aGC; (**b**) bar chart showing the mean fold changes in imGC, mGC and aGC following BIX-01294 treatment; (**c**) pie chart showing detailed number of upregulated genes in gametocytes following BIX-01294 treatment based on the predicted function; (**d**) genes 2-fold downregulated in aGC with high expression in gametocytes (GC) and/or ookinetes (OK) grouped by function (according to PlasmoDB; www.plasmodb.org).

In total, the transcripts of 359 genes were more than 1.5-fold de-regulated due to BIX-01294 treatment, 118 of which were upregulated, and 241 of which were downregulated. (Table S1). The majority of affected genes were identified in the activated gametocyte sample; here, 170 genes were transcriptionally downregulated, while, in mature gametocytes, only 26 genes exhibited a transcriptional de-regulation (Figure 4a). The mean fold changes ranged between 1.87 (immature gametocytes after 1 h of BIX-01294 incubation) and 0.49 (activated gametocytes after 1 h of BIX-01294 incubation) (Figure 4b). The three genes with upregulated transcript levels of more than 3-fold were identified in immature gametocytes and could be assigned to functions in transcription (TFIIS transcription factor PF3D7_0407300) and the U4 and U5 spliceosomal RNAs PF3D7_0308400 and PF3D7_1446000). The genes that exhibited the strongest downregulation effect (lower than 0.1-fold) were detected in activated gametocytes and encode putative exported proteins (the CX3CL1-binding protein CBP2 (GEXP07); PF3D7_1301700 and the unknown exported protein PF3D7_1149400) (Table S1). A high number of the downregulated genes could be assigned to transcription, translation, metabolism and proteostasis (Figure 4c). Other downregulated transcripts are associated with antigenic variation or encoding exported proteins, usually typical for the asexual blood stages. The majority of the downregulated genes are related to GO ontology biological processes such as reproduction, translation and regulation of immune system processes (Table S2). The majority of transcriptionally upregulated genes were of unknown functions (Figure 4c). Genes transcriptional upregulated in activated gametocytes were mainly predicted by GO ontology molecular function analyses to be associated with regulation of translation in response to stress, cell division and schizogony (Table S2).

As the strongest effect by BIX-01294 was observed for downregulated transcripts in activated gametocytes, we focused our subsequent analyses on genes with transcript levels lower than 2-fold that are known to have high expression levels in gametocytes and/or ookinetes (≥50% of peak expression as indicated at PlasmoDB, “Transcriptomes of seven sexual and asexual life stages” [6]). We found that the 45 identified genes could primarily be assigned to functions in transcription and translation as well as signaling and proteostasis (Figure 4d).

In accordance with the cell-based inhibition assays, the highest number of transcriptionally de-regulated genes were identified in activated gametocytes. The majority of these were transcriptionally downregulated due to BIX-01294 treatment, indicating that, under normal conditions, histone methylations promote expression of these genes. To be noted in this context, the main target of BIX-01294, G9a, thus far has been particularly assigned to histone methylation marks involved in gene repression, like H3K9 and H3K27 methylation [42,43]. In our study, however, BIX-01294 rather affected the methylation of H3K4, a mark that promotes gene expression in *Plasmodium* as well as in other organisms [24,38,44]. It is also probable that BIX-01294 may affect other histone methylations associated with the G9a HMT, such as H3K9me2 and H3K9me, which we did not investigate in the study.

The highest number of genes with peak expression levels during human-to-mosquito host transmission that were transcriptionally downregulated by BIX-01294 could be assigned to functions in translation. To be noted, the fine regulation of protein synthesis is of particular importance in activated gametocytes, since gametocyte activation triggers the synthesis of proteins required for the mosquito-specific stages [3]. The majority of the transcripts encoding such proteins are stored in the stress granules of female gametocytes, where they are translationally repressed by mRNA-binding ribonucleoproteins [3,45,46,47]

To validate the microarray array data, a total three genes, PF3D7_1466500, PF3D7_1250400 and PF3D7_0617800 transcriptionally upregulated in activated gametocytes following BIX-01294 treatment were selected. The activated gametocyte samples were treated with BIX-01294 at IC_90_ concentration or 0.5% vol. DMSO (untreated control) for 1 h and total RNA was isolated and cDNA synthesized. The synthesized cDNA was assessed for purity using stage specific markers by diagnostic RT-PCR (Figure S4a). A test for gDNA contamination in sample preparations lacking reverse transcriptase using *pfccp2*-specific primers was negative. Subsequently, the transcript expression levels of the three selected genes were measured via real time RT-PCR in BIX-01294 treated and untreated samples. Transcript expression was calculated by the 2^-ΔCt^ method [48] in which the threshold cycle number (Ct), was normalized to the Ct of the endogenous control gene encoding *P. falciparum* seryl tRNA-ligase (PF3D7_0717700) as reference gene. The results show that all three genes were upregulated following BIX-01294 treatment (Figure S4b).

Our combined data demonstrate the crucial role of histone methylations not only for asexual blood stage viability, but also for human-to-mosquito transmission of the malaria parasite. The plasmodial HMTs therefore represent prime multi-stage drug targets to kill the clinically relevant blood stages as well as the transmission-blocking stages of malaria parasites.

## 3. Materials and Methods

### 3.1. Antibodies

Antibodies used in this study were as follows: rabbit anti-histone H3 trimethyl K4 antibodies (H3K4me3) (Abcam, Cambridge, England, United Kingdomcity, state and country, ab213224), rabbit anti-H3K9me3 (Abcam, Cambridge, England, United Kingdom, ab8898), mouse anti-Pfs230 [4], mouse anti-Pf39 [49]. For indirect immunofluorescence assays, the following dilutions of the antibodies were used: anti-H3K4me3 (1:200), anti-H3K9me3 (1:200), mouse anti-Pfs230 (1:200). For Western blot analysis anti-H3K4me3, anti-H3K9me3 and anti-Pf39 were diluted 1:1000.

### 3.2. Parasite Culture

Parasites of the *P. falciparum* NF54 strain was cultivated in vitro in RPMI 1640 medium supplemented with 10% heat-inactivated human serum [50], and cultures were maintained at 37 °C in nitrogen containing 5% O_2_ and 5% CO_2_. Cultures were synchronized by repeated sorbitol treatment as described [51]. To induce gametocytogensis, lysed RBCs were added to cultures maintained at high parasitaemia. In order to kill the asexual blood stages in the gametocyte cultures, the cultures were supplemented with 50 mM N-acetyl glucosamine (GlcNac) for approximately 5 days once stage I gametocytes appear [52]. The gametocyte cultures were then maintained in normal culture medium without GlcNac until immature (stage II -IV) or mature stage V gametocytes were purified by Percoll gradient centrifugation [53]. Activated gametocytes were obtained after incubating Percoll-enriched mature gametocytes with 100 µM XA for 1 h at room temperature (RT). Purity of gametocyte samples were determined via Giemsa-staining. The human RBCs and sera used in this study were obtained from the Department of Transfusion Medicine (University Hospital Aachen, Aachen, Germany). The University Hospital Aachen Ethics commission approved all work with human blood (EK 007/13), the donors were anonymous and serum samples were pooled.

### 3.3. Malstat Assay

In order to determine the effect of the G9a histone methyltransferase inhibitor BIX-01294 (Sigma-Aldrich, St. Louis, Missouri, USA) on asexual blood stage replication, a Malstat assay was used as described previously [54,55]. Highly synchronized ring stage cultures were plated in 96-well plates (200 μL/well) in triplicate at a parasitaemia of 1% in the presence of BIX-01294 dissolved in 0.5% vol. DMSO at concentrations ranging from 5 µM to 0.06 nM. Chloroquine was used as internal positive control in the experiments while parasites incubated with DMSO (0.5% vol.) served as negative control. The parasites were cultured in vitro for 72 h, then resuspended and 20 μL was removed and added to 100 μL of the Malstat reagent in a 96-well microtiter plate. The parasite lactate dehydrogenase (pLDH) activity was then determined by adding 20 μL of a mixture of NBT (Nitro Blue Tetrazolium) and diaphorase (1:1; 1 mg/mL stock each) to the Malstat reaction, and the optical densities were measured at 630 nm. Each compound was tested 4 times, and the IC_50_ values were calculated from variable-slope sigmoidal dose-response curves using the GraphPad Prism program version 4 (GraphPad Software Inc., La Jolla, USA).

### 3.4. Gametocyte Toxicity Test

The toxic effect of the inhibitor on gametocytes was performed as described [5]. *P. falciparum* gametocyte producing NF54 strain parasites were culture at high parasitaemia to induce gametocytogenesis. Once mostly stage II gametocytes appear in the culture, 1 mL of culture was aliquoted in triplicate in a 24-well plate and treated with BIX-01294 at asexual blood stage IC_50_ (13 nM) and IC_90_ (0.12 µM) concentrations. Parasites treated with DMSO at a concentration of 1% vol. and chloroquine (IC_50_) were used as negative controls while the proteasome inhibitor epoxomicin (60 nM) served as a positive control in the experiments. The gametocytes were cultured for 10 d with daily medium change. The gametocytes were treated with the inhibitors for the first two days and there after the medium was inhibitor-free. Giemsa-stained blood smears were prepared every 2 d and the gametocytemia was determined by counting the numbers of gametocytes of stages II to V in a total number of 1000 erythrocytes in triplicate. The student’s *t*-test was used to determine significant differences between BIX-01294-treated and untreated samples.

### 3.5. Exflagellation Inhibition Assay

To determine the effect of BIX-01294 on the ability of male gametocytes to exflagellate, stage V mature gametocyte cultures were pre-incubated with BIX-01294 at concentrations ranging from 1 mM to 1 µM for 15 min at 37 °C. The samples were then activated with 100 μM XA at RT and after 15 min, the numbers of exflagellation centers were counted in 30 optical fields in duplicate using a Leica DMLS microscope at 400-fold magnification. The inhibition of exflagellation was then calculated as a percentage of the number of exflagellation centers in BIX-01294-treated cultures in relation to the number of exflagellation centers in untreated controls (untreated controls were set to 100%). TLCK (Tosyl-L-lysyl-chloromethane hydrochloride) at 30 μM was used as positive control in the experiments. The IC_50_ values were calculated from variable-slope sigmoidal dose-response curves using the GraphPad Prism program version 4 (GraphPad Software Inc., La Jolla, USA).

### 3.6. Macrogamete and Zygote Development Assays

The same volume of mature gametocyte cultures was treated with BIX-01294 at IC_50_ or IC_90_ concentrations or with 1% vol. DMSO as negative control for 1 h at 37 °C. Thereafter, the cultures were activated with 100 µM XA followed by incubation for 30 min (macrogamete development) or 12 h (zygote development) at RT. The samples were then coated on Teflon slides followed by immunolabelling with anti-Pfs25 as described below. The numbers of Pfs25-positive macrogametes or zygotes, characterized by their round shapes, were counted in a total number of 1000 erythrocytes. They were visualized by differential interference contrast and the slides were counted in triplicate using a Leica DMLS microscope at 1000-fold magnification. Zygotes were distinguished from macrogametes by their larger nuclei through Hoechst nuclear stain 3,3342 (Molecular Probes, Carlsbad, USA). The student´s *t*-test was used to determine significant differences between BIX-01294-treated and control samples.

### 3.7. Indirect Immunofluorescence Assay

Gametocyte cultures were coated on Teflon slides, allowed to air-dry and then fixed for 10 min in a methanol bath at –80 °C. The samples were afterwards incubated with 0.01% saponin/0.5% BSA/PBS and 1% neutral goat serum (Sigma-Aldrich, St. Louis, Missouri, USA)/PBS for 30 min at RT to permeabilize the parasite cell membrane and to block non-specific binding sites. Thereafter, the samples were incubated with the primary antibody diluted in 0.01% saponin/0.5% BSA/PBS for 1.5 h each at 37 °C. The primary antibody was visualized by incubating the samples with goat anti-mouse or anti-rabbit IgG secondary antibody conjugated to Alexa Fluor 488 (Molecular Probes, Carlsbad, USA) diluted in 0.01% saponin/0.5% BSA/PBS for 1 h at 37 °C. Double-labelling with stage-specific antibodies, i.e., Pfs230 and Pfs25 for the detection of gametocytes and activated gametocytes respectively was used. Nuclei were labeled by incubation with Hoechst nuclear stain 33342 for 1 min at RT and cells were mounted with anti-fading solution AF2 (Citifluor Ltd, Leicester, UK.) and sealed with nail varnish. Digital images were taken using a Leica AF 6000 microscope and processed using Adobe Photoshop CS software (Adobe, San Jose, Califonia, USA).

### 3.8. Western Blot Analysis

Immature gametocytes (stages II-IV), mature gametocytes (stage V) and 1 h post activated gametocytes were obtained as described above and haemoglobin was removed by lysing the RBCs with 0.05% saponin/PBS followed by a washing step with PBS. Parasite lysates were prepared by resuspending and sonicating the pellet in NP-40 lysis buffer (50 mM Tris HCl pH 8.0, 150 mM NaCl, 1% NP-40) containing protease inhibitor cocktail (Roche Diagnostics, Mannheim, Germany). The lysates were then resuspended in 1× SDS-PAGE loading buffer, denatured by heating for 10 min at 95 °C, and then separated via SDS-PAGE and transferred to Hybond ECL nitrocellulose membrane (Amersham Biosciences, New Jersey, USA) according to the manufacturer’s protocol. Non-specific binding sites were blocked by incubating membrane in Tris-buffered saline containing 5% BSA and 0.1% Tween 20. Afterwards, the membrane was incubated with the respective rabbit or mouse antibody for 2 h at RT. After washing, the membrane was incubated with anti-rabbit or anti-mouse IgG secondary antibody conjugated with alkaline phosphatase (Sigma-Aldrich, St. Louis, Missouri, USA) for 1 h at RT and developed in a solution of nitroblue tetrazolium chloride (NBT) and 5-bromo-4-chloro-3-indoxyl phosphate (BCIP; Sigma-Aldrich, St. Louis, Missouri, USA) diluted in water. Scanned blots were processed using Adobe Photoshop CS software.

### 3.9. Histone Methylation Detection

To investigate the effect of BIX-01294 on histone methylation, methylation assays were carried out to quantify H3K4me3 and H3K9me3 marks following treatment of gametocytes with BIX-01294. To this end, purified immature (stages II-IV), mature (stage V) gametocytes and 1 h post activated gametocytes were treated with BIX-01294 at IC_90_ concentrations or with 1% vol. DMSO (negative control) for 1 h and 6 h at 37 °C, respectively. Protein lysates were prepared and histone methylation was determined by Western blot analysis using anti-H3K4me3 and anti-H3K9me3 as described above. Anti-Pf39 was used as loading control. The histone methylation was quantified from 3 different experiments by measuring the band intensities via the ImageJ program, normalized to Pf39 and compared with respect to untreated samples

### 3.10. Microarray Analysis

Microarray experiments were performed as described previously [5]. Immature (stages II-IV) gametocytes, mature (stage V) gametocytes and gametocytes at 1 h post-activation following treatment with BIX-01294 at IC_90_ concentrations or with 0.5% vol. DMSO (untreated control) for 1 h and 6 h were obtained and total RNA was isolated using the Trizol reagent (Invitrogen, Carlsbad, USA) according to the manufacturer’s instructions. The quality of the RNA samples were determined using a ND-1000 (NanoDrop Technologies, Thermo Scientific, Waltham, USA) and by agarose gel electrophoresis. The cDNA synthesis, labeling and hybridization experiments were carried out as described previously [41]. Briefly, amino-allyl cDNA synthesis was performed using Superscript II reverse transcriptase (Invitrogen, Carlsbad, USA). The amino-allyl cDNA was then purified and concentrated using the Zymo DNA clean and concentrator-5 column (Zymo Research, California, USA). This was then followed by sample coupling using the Cy5 dye (GE Healthcare, Chicago, USA). A reference pool consisting of a mixture of RNA from asexual blood stages and gametocytes was also prepared and labelled with the Cy3 as described above. The same amounts of Cy5-labelled samples from each treatment and Cy3-labelled reference pool were subjected to array hybridization for 17 h at 65 °C using a *P. falciparum* DNA Agilent microarray chip (Agilent Technologies AMADID #037237) containing the 5363 coding genes [40]. The arrays were scanned using the Agilent G2505B microarray scanner (Agilent Technologies,Santa Clara, USA). The Agilent feature extractor software version 9.5 was then used to extract normalized intensities and they were uploaded to the Princeton University Microarray Database (PUMA.princeton.edu) for analysis. Following background subtraction, the log_2_ of the (Cy5/Cy3) intensity ratio was extracted and the transcript abundance of BIX-01294-treated samples were compared to that of untreated samples. The raw intensity data have been submitted to the NCBI Gene Expression Omnibus (GEO; http://www.ncbi.nlm.nih.gov/geo/) under accession number GSE136008. For the selection of up- and downregulated genes, a cut-off value of greater than 1.5-fold for at least one of the two time-points with a consistent up- or downregulation for both time-points was considered significant. Data were analysed using Microsoft Excel 2010. The data base PlasmoDB (http://plasmodb.org/plasmo) was used for gene annotation analyses and GO ontology analysis.

### 3.11. Semi- Quantitative and Real Time RT-PCR

To determine the transcript expression of the 10 SET-domain containing HMTs, total RNA was isolated from trophozoites, schizonts, immature, mature and 30 min post activated gametocytes as described above. For microarray validation, RNA was isolated from enriched gametocytes activated for 1 h followed by treatment with BIX-01294 at IC_90_ concentration or with 0.5% vol. DMSO (untreated control) for 1 h. In both experiments, one µg of total RNA from each sample was reverse transcribed into cDNA using the SuperScript IV First-Strand Synthesis System (Invitrogen, Carlsbad, USA), following the manufacturer’s instructions. To determine if the gametocyte samples were not contaminated with asexual blood stages, the cDNA was tested by diagnostic PCR using primers specific for the gene encoding the apical membrane antigen AMA-1 (asexual blood stage contamination) and for gametocyte-specificity using primers specific for the gene encoding the LCCL-domain protein PfCCp2 [4,5]. To investigate potential gDNA contamination, controls without reverse transcriptase were also used. Primers for quantitative real time and semi quantitative RT–PCR were designed using the online tool Primer 3 (http://frodo.wi.mit.edu/primer3/) and tested on gDNA or cDNA in PCR to confirm primer specificity (for primer sequences, see Table S3). Real-time RT-PCR measurements were performed using the Step One Plus Real-Time Detection System (Thermo Scientific, Waltham, USA). Reactions were performed in triplicate in a total volume of 20 μL using the maxima SyBR green qPCR master mix according to manufacturer’s instructions (Thermo Scientific, Thermo Scientific, Waltham, USA). Controls without template and without reverse transcriptase were also included in all real time RT-PCR experiments. Transcript expression levels were calculated by the 2^-ΔCt^ method [48] using the endogenous control gene encoding the *P. falciparum* seryl tRNA-ligase (PF3D7_0717700) as references [4,5]. For semi-quantitative PCR, the following PCR cycling conditions were used: an initial denaturation step at 94°C for 2 min, followed by 25 cycles of denaturation at 94 °C for 15 s, annealing at 45°C (for 30 s, extension at 72 °C for 45 s, followed by a final extension at 72 °C for 2 min.

## 4. Conclusions

Our combined data highlight the importance of histone methylation in both the asexual blood stages as well as the sexual stages of the malaria parasite *P. falciparum* thereby confirming that histone methyltransferase inhibitors could be exploited as multi-stage drugs to kill the parasite.

## Supplementary Materials

Supplementary materials can be found at https://www.mdpi.com/1422-0067/20/20/5087/s1.

## Abbreviations

aGC	Activated gametocytes
GC	Gametocytes
HAT	Histone acetyltransferase
LD	Linear dichroism
HDAC	Histone deacetylase
HDM	Histone demethylase
HMT	Histone methyl transferase
imGC	Immature gametocytes
mGC	Mature gametocytes
NRS	Normal rabbit serum
TSA	Trischostatin A
XA	Xanthurenic acid
