# Supplementary material for: The G9a Histone Methyltransferase Inhibitor BIX-01294 Modulates Gene Expression during Plasmodium falciparum Gametocyte Development and Transmission
Source: Int J Mol Sci. 2019 Oct 14;20(20):5087. doi: 10.3390/ijms20205087 (PMC6829282; doi:10.3390/ijms20205087)
Supplement: Supplementary file 1 [file ijms-20-05087-s001.zip › IJMS-Supplementary-for proof/Ngwa et al., 2019 Figure supplements-Rev.pptx]

## Slide 1
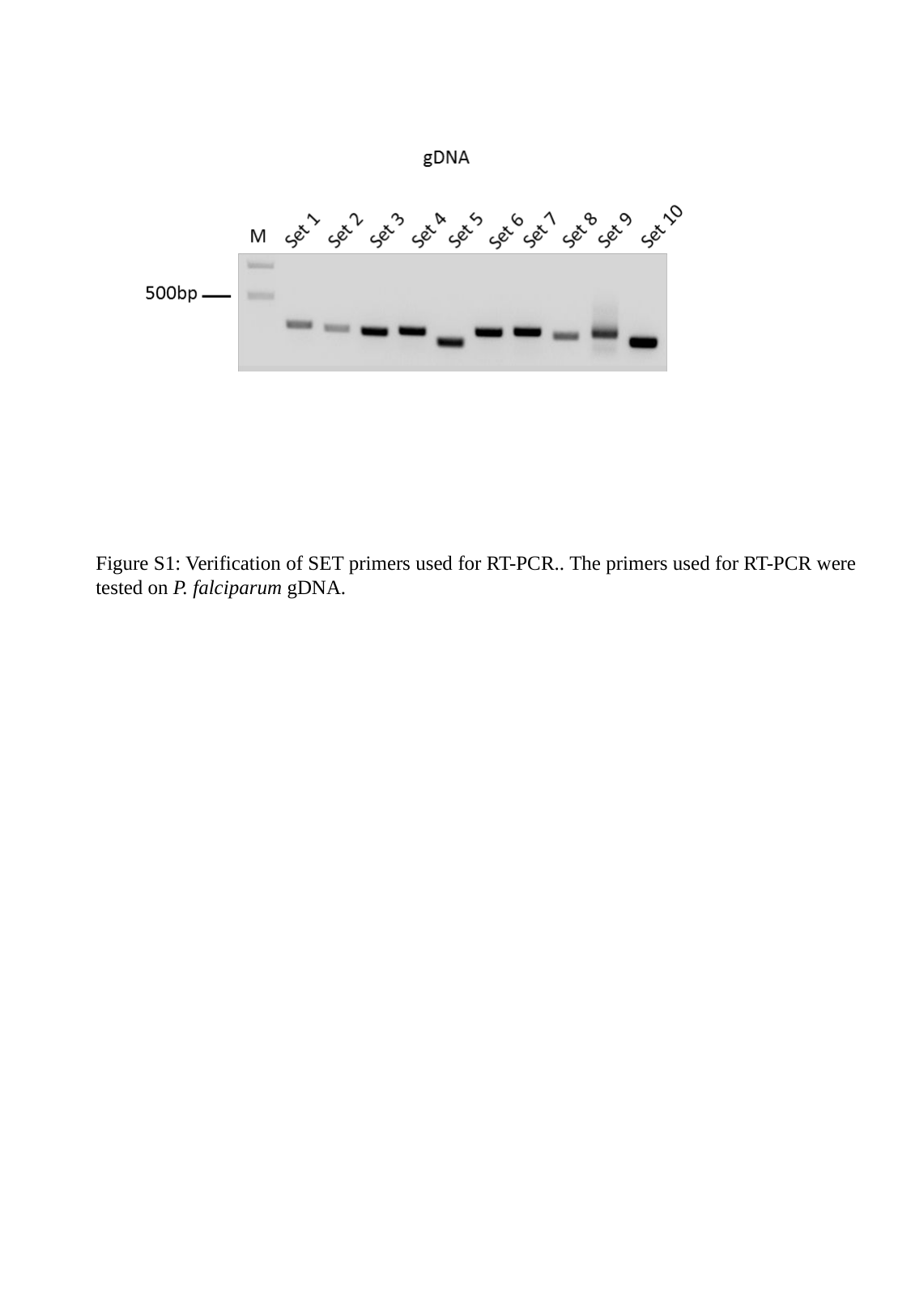

Figure S1: Verification of SET primers used for RT-PCR.. The primers used for RT-PCR were tested on P. falciparum gDNA.

## Slide 2
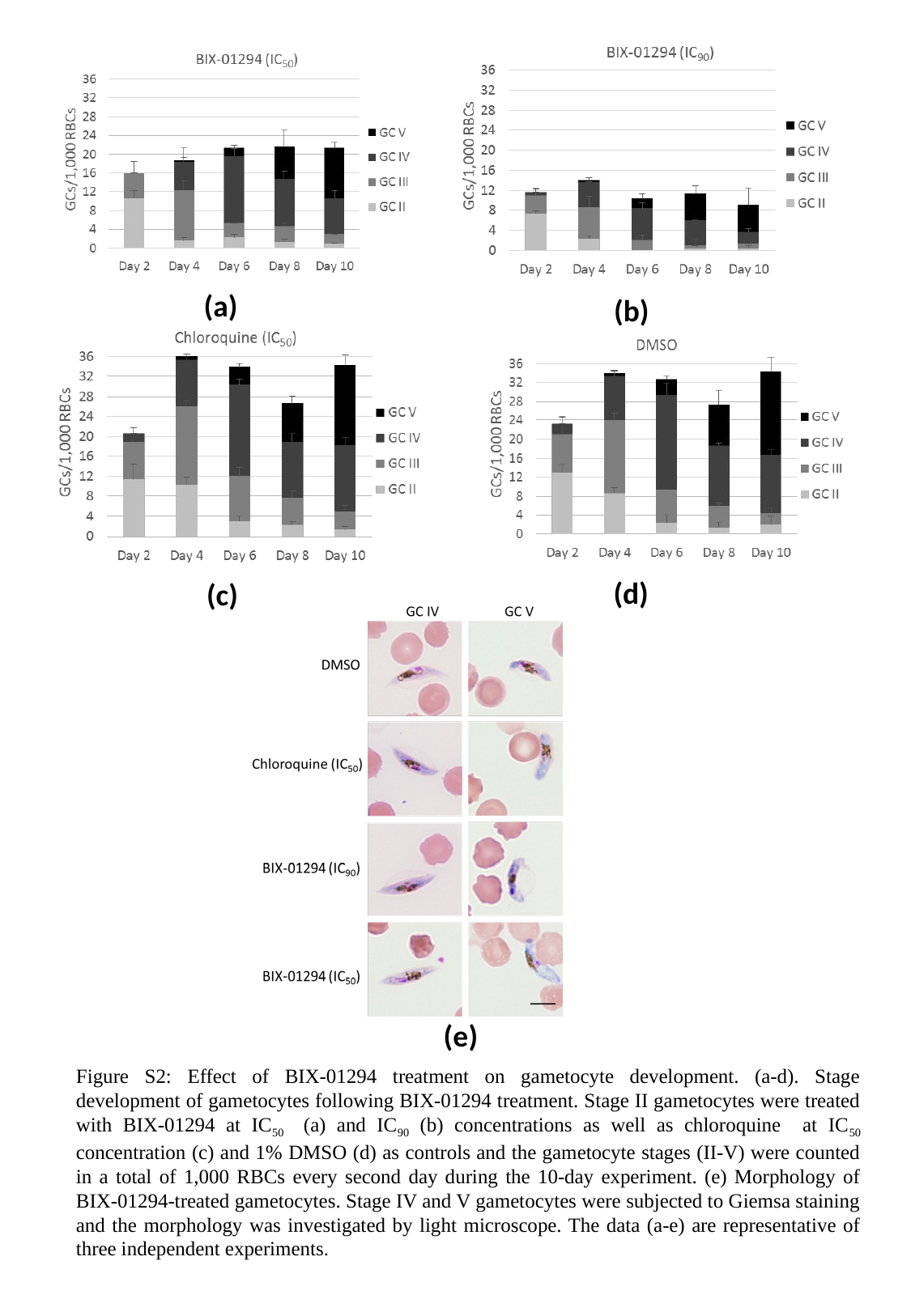

(a)
(b)
(d)
(c)
(e)
Figure S2: Effect of BIX-01294 treatment on gametocyte development. (a-d). Stage development of gametocytes following BIX-01294 treatment. Stage II gametocytes were treated with BIX-01294 at IC50 (a) and IC90 (b) concentrations as well as chloroquine at IC50 concentration (c) and 1% DMSO (d) as controls and the gametocyte stages (II-V) were counted in a total of 1,000 RBCs every second day during the 10-day experiment. (e) Morphology of BIX-01294-treated gametocytes. Stage IV and V gametocytes were subjected to Giemsa staining and the morphology was investigated by light microscope. The data (a-e) are representative of three independent experiments.

## Slide 3
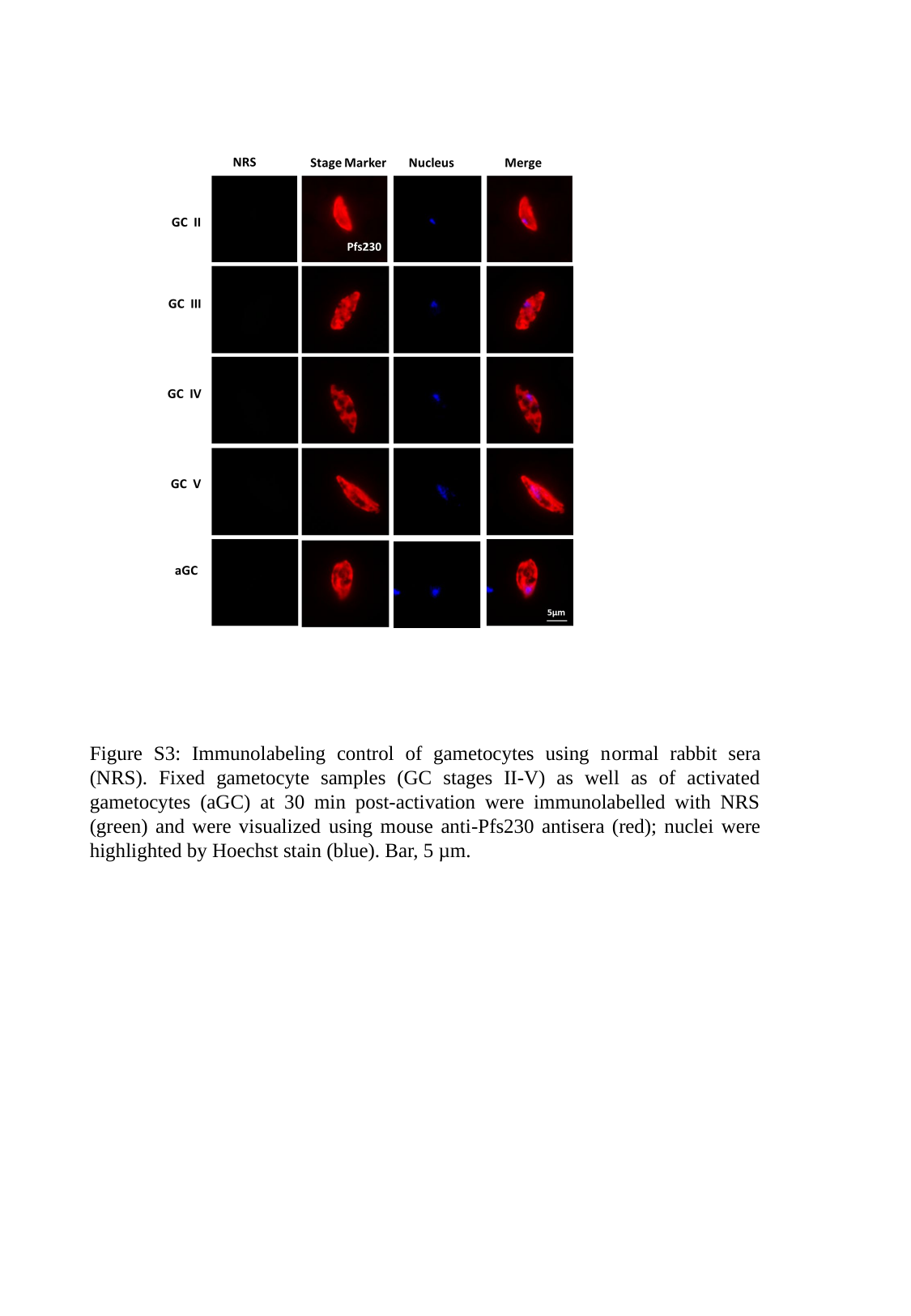

Figure S3: Immunolabeling control of gametocytes using normal rabbit sera (NRS). Fixed gametocyte samples (GC stages II-V) as well as of activated gametocytes (aGC) at 30 min post-activation were immunolabelled with NRS (green) and were visualized using mouse anti-Pfs230 antisera (red); nuclei were highlighted by Hoechst stain (blue). Bar, 5 µm.

## Slide 4
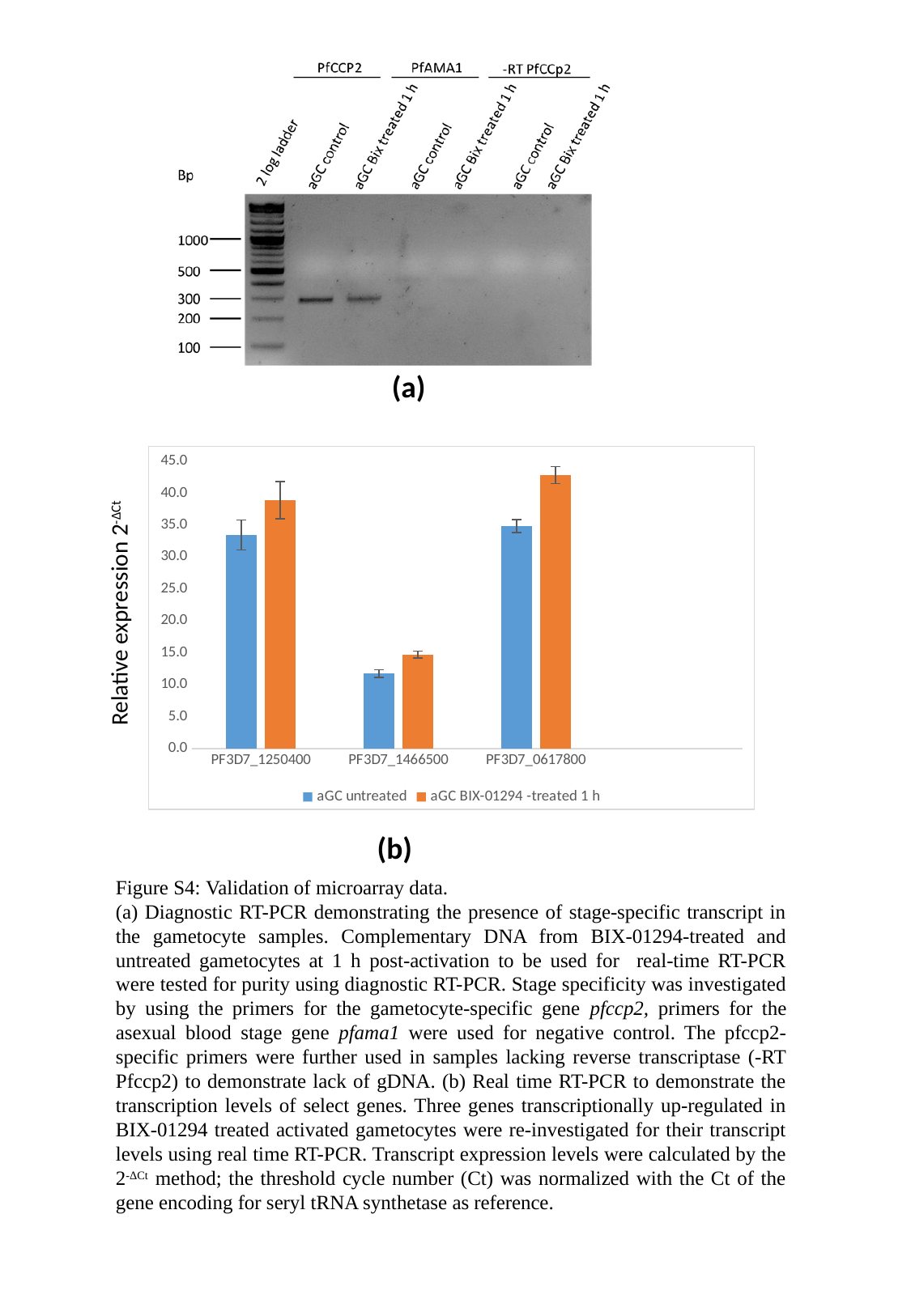

(a)
### Chart
| Category | aGC untreated | aGC BIX-01294 -treated 1 h |
|---|---|---|
| PF3D7_1250400 | 33.461119665949106 | 38.908778349069365 |
| PF3D7_1466500 | 11.748465130805618 | 14.724452575413638 |
| PF3D7_0617800 | 34.85100805900853 | 42.846365398838266 |Relative expression 2-ΔCt
(b)
Figure S4: Validation of microarray data.
(a) Diagnostic RT-PCR demonstrating the presence of stage-specific transcript in the gametocyte samples. Complementary DNA from BIX-01294-treated and untreated gametocytes at 1 h post-activation to be used for real-time RT-PCR were tested for purity using diagnostic RT-PCR. Stage specificity was investigated by using the primers for the gametocyte-specific gene pfccp2, primers for the asexual blood stage gene pfama1 were used for negative control. The pfccp2-specific primers were further used in samples lacking reverse transcriptase (-RT Pfccp2) to demonstrate lack of gDNA. (b) Real time RT-PCR to demonstrate the transcription levels of select genes. Three genes transcriptionally up-regulated in BIX-01294 treated activated gametocytes were re-investigated for their transcript levels using real time RT-PCR. Transcript expression levels were calculated by the 2-ΔCt method; the threshold cycle number (Ct) was normalized with the Ct of the gene encoding for seryl tRNA synthetase as reference.
