# Supplementary material for: The G9a Histone Methyltransferase Inhibitor BIX-01294 Modulates Gene Expression during Plasmodium falciparum Gametocyte Development and Transmission
Source: Int J Mol Sci. 2019 Oct 14;20(20):5087. doi: 10.3390/ijms20205087 (PMC6829282; doi:10.3390/ijms20205087)
Supplement: Supplementary file 1 [file ijms-20-05087-s001.zip › IJMS-Supplementary-for proof/Table S3 primer list.pdf]

Table 3. List of primers used in the study.

**Primers for Semi-Quantitative RT PCR**	
Set1-RT S	TTGTCCGATGCTATGAATGG
Set1-RT AS	GCGCATTCGTATCCAAATTA
Set2-RT S	GTTCTTTGCCGTTTTTGTCA
Set2-RT AS	TTTGTGTTATGCGCATGGAT
Set3-RT S	CGAAGGGAGAGGTGTCGTAA
Set3-RT AS	AGGCTCATCCACAAAATCGT
Set4-RT S	ATTTCGACCTGCTTCAATGC
Set4-RT AS	AAGAAACATCCCAGGAACCA
Set5-RT S	GGAAAATCCTCTCTTGGTGGT
Set5-RT AS	AATCGACGAGGTTTCTTGGT
Set6-RT S	CGAGCAGGATATTGCTTGGT
Set6-RT AS	CCAAGCTCGCTCTAAACACA
Set7-RT S	CCAAGTGCAATGTTTGCCTA
Set7-RT AS	TTGCTTTTTCCCATTGAGATG
Set8-RT S	TATTGACCGCCACAACAATG
Set8-RT AS	TCGGCGTTTCTTTATTTTGC
Set9-RT S	GAAAATGAATCGGTGGATGG
Set9-RT AS	CACCGTTTGTATCGTCACCA
Set10-RT S	TGAAATTAATCCCGGTGAGG
Set10-RT AS	TCCACAATATCCTCCATTCCA
**Primers for Microarray Validation**	
PF3D7_1466500 S	CCATTAAGAAGATGGCAGGTG
PF3D7_1466500 AS	CCATTCACATCAATTGTTTTATCA
PF3D7_1250400 S	CAGAATGAAAAGTGTACCATGACA
PF3D7_1250400 AS	TGGTTCGTTTCCTCATCTTTC
PF3D7_0617800 S	GCCTCAAAGGGAACTTCAAA
PF3D7_0617800 AS	TCTTGCTGCATTTCCTGCTA
